# The Inconveniences of the Plaster Jacket and How to Avoid Them

**Published:** 1879-04

**Authors:** Edmund Andrews

**Affiliations:** No. 6 16th St., Chicago


### ARTICLE IV.

### THE INCONVENIENCES OF THE PLASTER JACKET, AND HOW TO AVOID THEM. By EDMUND ANDREWS, A.M., M.D.

One evil of the plaster of Paris jacket is the impossibility of removing it for the purpose of bathing. Perspiration often accumulates underneath it, and becomes very offensive and adapted to generate disease, so that it becomes necessary, if the patient's life is to be that of a civilized human being, to construct a new jacket in from two to four weeks.

Another trouble is the impossibility of varying the tightness of the appliance to suit the varying plumpness or thinness of the patient.

A third trouble is the disagreeable suspension of the body by the head and shoulders, the frequent repetition of which awakens great dread in the minds of some patients. A few practical remarks on the efforts which have been made to overcome these difficulities, will be of great use to many members of the profession.

In order to produce an apparatus that can be removed at pleasure, for bathing and other purposes, and which can be rendered tight or loose at will, Prof. Yandell, of Louisville, has constructed the following apparatus: A thin plaster of Paris jacket is first applied in the ordinary way. This is immediately removed and made use of merely as a mould from which to cast a fac simile in plaster of the patient's body. Around this model he then winds alternate layers of cloth bandage and manilla paper dipped in glue. After this has been laid on to the thickness of 3 or 4 mm., it is allowed to dry into a solid mass. A strip about 5 centimeters wide is then removed from the median line in front, and the whole finished by gluing in a soft lining, and inserting eyelets or hooks for lacing. The elasticity of the material allows this apparatus to be taken from the body at pleasure, by simply springing it open in front. When laced up it has all the rigidity of the plaster jacket, and it can be brought to any degree of tightness.

To avoid the destruction of the apparatus by the strain upon its texture in springing it open, Dr. Chas. Fegers, of McHenry, Ills, has devised a similar jacket, but with an opening both before and behind. The posterior one, about 3 centimeters wide, is closed with cloth, free from glue, which extending around the sides encloses between its layers the more inflexible material, and firmly adheres to them. In front the jacket is closed by lacing strings running through eyelets or hooks, as in Prof. Yandell's jacket. This furnishes a perfectly flexible cloth hinge down the back, which enables the apparatns to be opened and closed more easily than the other. Dr. Fegers has applied his apparatus to a number of cases with excellent results.

Suspension by the head and shoulders is extremely disagreeable, and even painful to some persons. It involves the risk, too, that should the patient present one of those cases of relaxed cervical articulation, which sometimes exist, the strain on the hooks might result in the fearful disaster of a dislocated neck.

Besides, the suspension apparatus is cumbersome and not generally possessed by the physician.

All these inconviences and risks can be avoided, and the best possible position for the patient secured in a very simple manner, and without any apparatus whatever.

In case of a child, if one assistant support the inferior extremities, and the other the head and shoulders, and hold the body in a horizontal position with the face downwards, the center of the spine will fall and tend to straighten the curve, and accomplish the desired extension as perfectly as can be done with the suspension apparatus; the patient being held in this position, the surgeon can apply the plaster bandage in the usual way, and then remove it by dividing in the back or front, and use it for a mould for the construction of a Yandell's or Feger's jacket, as above mentioned.

In case of an adult patient, two tables can be placed end to end with space between them equal to the length of the patient's trunk, pillows being placed beneath his shoulders and hips; he is laid face downward with the trunk extending across the space between the tables; an assistant helping to maintain the position of the patient at either end. The surgeon may now apply the jacket just as before detailed. From a considerable experience, I am prepared to assert that the results obtained by this method of horizontal suspension are in every way equal to those following the perpendicular hanging, while the process itself is simple and far less disagreeable to the patient.

The plan of taking moulds for spinal cases in the prone, horizontal position with the chief support at the hips and shoulders, so as to allow the body between to settle downward, and thus correct the curvature, was introduced into Chicago some ten years ago, by the late Prof. Sherman, as a means of preparing his leather backed corset-supporter. Prof. Sayre afterwards employed a similar position in what he called the "turtle shell" plan; and probably many others have resorted to such an obvious method of fitting aparatus, so that one cannot readily settle the question of priority. Of late years the suspension plan has become fashionable, and is almost exclusively used at the present time.

The difficulties and possible dangers of the perpendicular hanging process appear a little formidable to practitioners who are unaccustomed to it, but the simplicity and comparative comfort of the horizontal suspension is such that any ordinary physician can undertake it without hesitation.

No. 6 16th St., Chicago, March 13, 1879.

---

PICTURES FROM THE PARISIAN HOSPITAL.—Professor (who has his class in the wards) to patient, "What is your occupation?" Patient (who has pulmonary disease), "Musician, sir." Professor, to class: "There, gentlemen, at last I have the opportunity of demonstrating what I have often told you in the lecture room, that the wear and tear on the respiratory tract caused by the blowing of musical instruments, is a fertile source of just such difficulty as our patient here labors under. (To patient) What instrument do you play, sir?" Patient: "The bass drum!"

MILK-TEST.—A German paper gives a test for watered milk which is simplicity itself. A well polished knitting needle is dipped into a deep vessel of milk and immediately withdrawn in an upright position. If the sample is pure, some of the fluid will hang to the needle; but if even a small proportion of water has been added to the milk, the fluid will not so adhere.—*Med. Record.*

COMA AND ALCOHOL.—Dr. Macewen, of Glasgow, lays down the rule that an insensible person who, being left undisturbed for from ten to thirty minutes, has contracted pupils, which dilate on his being shaken, without any return of consciousness, and then contract again, can be laboring under no other condition than alcoholic coma.

IN A discussion on cremation at a London club, a member is credited with the argument, "We *earn* our living; why should we not *urn* our dead?"

